# Persistent Exposure to Environmental Levels of Microcystin-LR Disturbs Cortisol Production via Hypothalamic-Pituitary-Interrenal (HPI) Axis and Subsequently Liver Glucose Metabolism in Adult Male Zebrafish (*Danio rerio*)

**DOI:** 10.3390/toxins12050282

**Published:** 2020-04-28

**Authors:** Lingkai Wang, Wang Lin, Qingji Zha, Honghui Guo, Dandan Zhang, Liping Yang, Li Li, Dapeng Li, Rong Tang

**Affiliations:** 1 College of Fisheries, Huazhong Agricultural University, Wuhan 430070, China; 2 Hubei Provincial Engineering Laboratory for Pond Aquaculture (Huazhong Agricultural University), Wuhan 430070, China; 3 National Demonstration Center for Experimental Aquaculture Education (Huazhong Agricultural University), Wuhan 430070, China

**Keywords:** microcystin-LR, hypothalamic-pituitary-interrenal axis, cortisol, liver glucose metabolism, zebrafish

## Abstract

There is growing evidence that microcystin-LR (MC-LR) is a new endocrine disruptor, whereas the impacts of persistent exposure to MC-LR on the hypothalamic-pituitary-interrenal (HPI) axis and health hazards thereafter have not been investigated. In this work, adult male zebrafish (*Danio rerio*) were immersed into MC-LR solutions at concentrations of 0, 1, 5 and 25 μg/L for 30 d, respectively. The results showed that persistent MC-LR exposure caused an extensive upregulation of HPI-axis genes but an inhibition of brain nuclear receptors (*gr* and *mr*), which finally increased serum cortisol levels. Furthermore, the decreased expression of hepatic *gr* might partly be responsible for the strong inhibition on the expression of downstream genes involved in glucose metabolic enzymes, including gluconeogenesis-related genes (*pepck*, *fbp1a*, *g6pca*), glycogenolysis-related gene (*pyg*), glycolysis-related genes (*gk*, *pfk1b*, *pk*) and glycogenesis-related gene (*gys2*). These findings are in accordance with the decline in serum glucose, indicating that long-term MC-LR exposure caused a lower production of glucose relative to glucose lysis. Our above results firstly establish the link between persistent MC-LR exposure and impaired glucose metabolism, suggesting that long-term MC-LR-mediated stress might threaten fish’s health.

## 1. Introduction

Due to water eutrophication and global warming, the high frequencies of cyanobacterial blooms have attracted growing interest in recent decades [1,2,3]. Microcystins (MCs), a group of cyclic peptides with seven amino acids, are the most commonly detected cyanotoxins in freshwater blooms. At present, 279 structurally related analogues of MCs have been recognized [4]. Microcystin-LR (MC-LR) is the most widely distributed, abundant and toxic among these congeners [5]. The level of 1 μg/L MC-LR is recommended as the standard concentration in drinking water by the World Health Organization [6]. However, the real concentration of dissolved MC-LR in natural water is in the range of 0.1–10 μg/L, which generally oversteps the recommended level and causes potential harm to aquatic animals and human beings [7,8].

Exposure to MCs has been shown to result in multiorgan toxicity, such as hepatotoxicity [9], nephrotoxicity [1], cardiac toxicity [10], splenic toxicity [11] and neurotoxicity [12]. Recent studies have shown that MC-LR can affect the reproductive endocrine functions of fish, which are characterized with altered sex hormone levels and transcriptional profiles of genes, along the hypothalamic-pituitary-gonad (HPG) axis [13,14,15,16]. Hou et al. [17] revealed that MC-LR could elicit estrogenic responses via disrupting steroidogenesis. It has been found that MC-LR can induce thyroid dysfunction in fish through the regulation of the hypothalamic-pituitary-thyroid (HPT) axis [18,19]. These prior studies suggested that MC-LR could serve on an endocrine disruptor. In fish, the endocrine system consists of three main pathways, including the HPG axis, HPT axis and hypothalamic-pituitary-interrenal (HPI) axis. The effects of chemicals on an endocrine axis pathway could indirectly influence the other endocrine axes of fish [20]. This is corroborated by a prior study result of Bury et al. [21], who reported that acute exposure to lysed *Microcystis aeruginosa* cells caused an increase in the level of plasma cortisol, one of the HPI-axis final effectors, in brown trout *Salmo trutta*. Li et al. [22] also demonstrated that plasma cortisol levels were significantly elevated in crucian carp, injected intraperitoneally with *Microcystis* extract corresponding to 50 and 200 μg/kg body weight of puried MC-LR, implying that MC-LR might be capable of disrupting the adrenal endocrine system. Nevertheless, the present information on the impacts of MC-LR on the HPI axis is very limited in contrast to that of the HPG and HPT axes. 

Cortisol is the most abundant and active corticosteroid in teleost fish, which plays crucial roles in controlling whole body homeostasis and response to the stress [23]. By coping with stress, cortisol levels are often elevated, which subsequently interferes with carbohydrate homeostasis, so as to meet higher energy demand [23,24,25]. In previous documents, MCs have been shown to result in disturbances of plasma glucose levels in fish [21,26,27,28]. Several omics investigations also show a positive association between MCs and abnormal carbohydrate metabolism [29,30]. It is noteworthy that teleost fish are glucose intolerant in general. Impaired glucose metabolism therefore elicits persistent hyperglycemia or subsequent physiological disturbances [31]. However, how cortisol is involved in the modulation of hepatic glucose metabolism disorder induced by MC-LR in fish and the underlying mechanism is still unclear. 

Zebrafish, a kind of model organism, has been widely used to investigate chemical toxicity for its short life cycle, rapid development and growth, and the fact that it is easy to culture. In addition, prior MC-LR studies have focused primarily on high-dose acute treatments, while longer-term lower doses of MCs exposure appear to be more common and realistic than acute intoxications. The aim of the present study was therefore, through a persistent exposure experiment where adult zebrafish were immersed in MC-LR solutions at environmental equivalent levels of 0, 1, 5, 25 μg/L for 30 d, to evaluate the toxic effects of MC-LR on corticosteroid production via HPI axis and to discuss the underlying mechanism of corticosteroid influencing MC-LR-induced abnormal liver glucose metabolism. 

## 2. Results

### 2.1. Serum Cortisol and Glucose Levels

**Figure 1:**
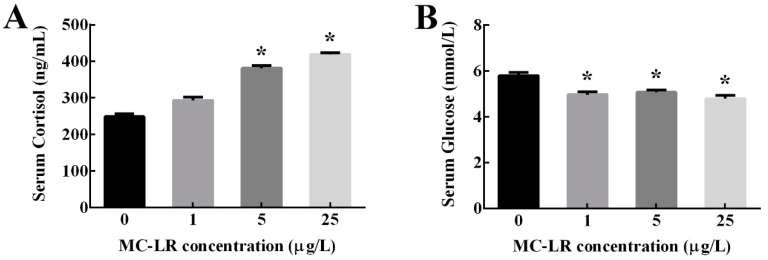
The contents of serum cortisol (**A**) and glucose (**B**) in male zebrafish after a 30-d exposure to microcystin-LR (MC-LR) at different concentrations of 0, 1, 5 and 25 μg/L, respectively. Data are shown as mean ± SE (n = 3). Asterisk (*) indicates significant differences at *p* < 0.05 between MC-LR treatments and the control.

As shown in Figure 1, serum cortisol levels were significantly increased in 5 and 25 μg/L MC-LR treatment groups compared with the control group (*p* < 0.05). Conversely, serum glucose levels were significantly decreased in three MC-LR treatment groups (*p* < 0.05).

### 2.2. The Expression of Genes Related to HPI Axis

**Figure 2:**
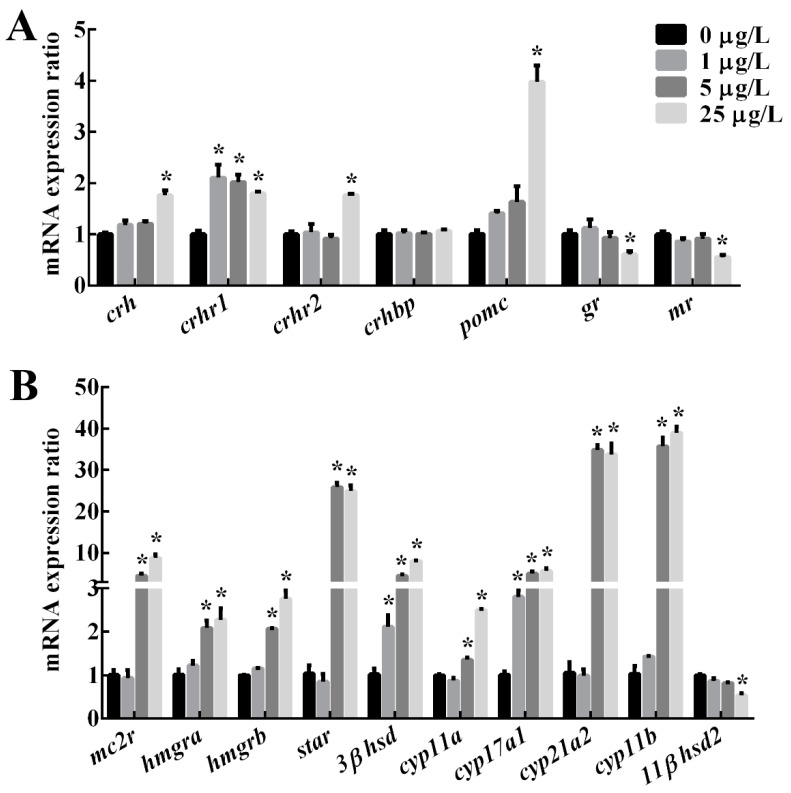
Relative mRNA expression of genes related to hypothalamic-pituitary-interrenal (HPI) axis in the brain (**A**) and interrenal (**B**) of adult male zebrafish after a 30-d exposure to MC-LR at different concentrations of 0, 1, 5 and 25 μg/L, respectively. Values are reported as mean ± S.E. (n = 6). Asterisk (*) indicates significant differences at *p* < 0.05 between MC-LR treatments and the control.

The effects of MC-LR treatment on the relative expression of genes involved in the HPI axis were shown in Figure 2 and Table 1. In the brain, mRNA levels of *crh*, *crhr2* and *pomc* were significantly upregulated in the 25 μg/L MC-LR group, and *crhr1* mRNA were significantly increased in all MC-LR treatment groups (*p* < 0.05). Additionally, the expression of *crhbp* was not affected significantly in any treatment group. In contrast, mRNA levels of *gr* and *mr* were significantly down-regulated in 25 μg/L MC-LR groups (*p* < 0.05). 

In the head kidney, the expression of *mc2r*, *star*, *cyp11a*, *cyp21a2* and *cyp11b* was significantly elevated in 5 and 25 μg/L MC-LR groups (*p* < 0.05). Moreover, the expression levels of *3βhsd* and *cyp17a1* were significantly up-regulated by all MC-LR treatments (*p* < 0.05), while the expression of *11βhsd2* was significantly down-regulated in the 5 and 25 μg/L MC-LR groups (*p* < 0.05). 

### 2.3. The Expression of Genes Related to Hepatic Glucose Metabolic Enzymes

**Figure 3:**
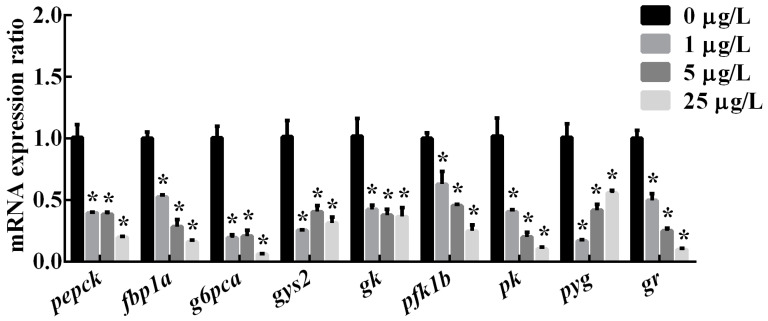
The relative mRNA expression of genes related to hepatic glucose metabolic enzymes in adult male zebrafish after a 30-d exposure to MC-LR at different concentrations of 0, 1, 5 and 25 μg/L, respectively. Values are reported as (mean ± S.E., n = 6). Asterisk (*) indicates significant differences at *P*< 0.05 between MC-LR treatments and the control.

**Figure 4:**
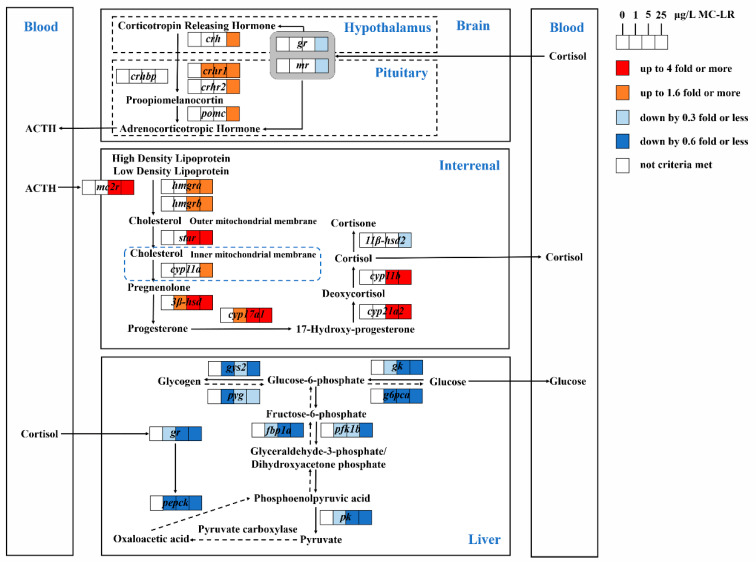
Graphical summary of the impacts of MC-LR on the HPI axis and thereafter hepatic glucose metabolism disorder in male zebrafish.

In the liver, mRNA levels of glycolysis-related genes (*gk*, *pfk1b*, *pk*) and the glycogenolysis-related gene (*pyg*) were significantly downregulated in three MC-LR treatment groups (Figure 3 and Figure 4). Similarly, gluconeogenesis-related genes (*pepck*, *fbp1a*, *g6pca*) and the glycogenesis-related gene (*gys2*) were significantly decreased by all MC-LR treatments (*p* < 0.05). The expression of gene *gr* in the liver of male zebrafish was also down-regulated significantly in the 1, 5 and 25 μg/L MC-LR groups (*p* < 0.05).

## 3. Discussion

Recently, the disruption of MCs on the endocrine system of fish has been the focus of the risk assessment of the ecological environment harm of toxic cyanobacterial blooms [17,19], whereas the impacts of chronic exposure to MCs on the HPI axis and thereafter health hazards have not been investigated. This present study firstly revealed that persistent exposure to environmental levels of MC-LR interferes with interrenal cortisol production via modulating the expression of HPI-axis genes, which subsequently impaired hepatic glucose metabolism and posed a threat to fish’s health.

Experimental data have shown that toxicants can elicit a stress response in teleosts, where HPI axis is activated and stress hormones are produced in response to the disturbance and restoring homeostasis [32,33,34]. In general, the activation of the HPI axis begins with the release of corticotropin-releasing hormone (CRH) from the hypothalamus, which thereafter triggers the secretion of the adrenocorticotropic hormone (ACTH) in the pituitary. Meanwhile, the CRH signal is modulated by receptors (CRHR1, CRHR2) and a binding protein (CRHBP) [35]. Circulating ACTH binds to the melanocortin type 2 receptor (MC2R) in the interrenal cells of head kidney and stimulates the conversion from high-density lipoprotein and/or low-density lipoprotein to cholesterol. After crossing the outer mitochondrial membrane, cholesterol is then transported to the inner mitochondrial membrane via the steroidogenic acute regulatory protein (StAR). Through a series of enzymatic reactions, cortisol is synthesized in the interrenal tissue of the head kidney and released into the circulating blood. Thus, the level of circulating cortisol, as a final product of the HPI axis, is usually used as an important indicator of the degree of stress response in teleost fish. Previous studies involving short-term exposure to MCs have showed that such exposures lead to disturbed physiology in fish, which are characterized by increased blood cortisol [21,22]. In this study, after 30 d of exposure, zebrafish treated with 5 and 25 μg/L MC-LR showed elevated serum cortisol levels relative to the controls, suggesting that fish suffered a continued physiological stress. In accordance with increased circulating cortisol levels, the transcription levels of HPI-axis genes except brain *gr* and *mr* as well as interrenal *11βhsd2* were significantly increased in zebrafish, following exposure to MC-LR. Several studies have documented that MC-LR can be accumulated in the brains of fish and cause neuronal histological damage and development toxicity [2,19,36]. It is then not surprising to detect the alterations of relevant HPI-axis genes in the zebrafish brain induced by MC-LR since the brain is the center of neuroendocrinology. Marked up-regulation of the primary neurohormone, CRH, was also reported in zebrafish larvae exposed to 500 μg/L MC-LR for 96 h [37] and in juvenile zebrafish after exposure to 25 μg/L MC-LR for 7, 14, 21 or 28 d [38]. Similar adverse effects on brain CRH contents were observed in studies on other chemicals exposure such as prochloraz [20] and metyrapone [34]. It was indicated by our finding, together with prior studies, that MC-LR might directly disrupt the structure and function of brain tissue and lead to subsequent corticosteroid signaling disruption. To our knowledge, the interrenal gland of teleosts is the site involved in the production of cortisol. STAR, as the rate-limiting enzyme, modulates the transportation of cholesterol [32]. CYP11A and CYP11B regulate the first and end steps in the corticosteroido-genesis pathway, respectively [39,40]. In the present study, these three key enzymes genes (*star*, *cyp11a* and *cyp11b*) in the interrenal tissue were significantly up-regulated in fish treated with 5 and 25 μg/L MC-LR, indicating that persistent exposure to MC-LR affected the synthesis of interrenal cholesterol. The similar up-regulation of *star* and *cyp11a* were reported in zebrafish gonad [17], where environmental concentrations of MC-LR were proven to increase the synthesis of sex steroid hormone via the regulation of the HPG-axis. In fact, in vertebrates, the brain and adrenal/interrenal tissue, as well as the gonad, share parts of steroidogenesis-related genes like *star*, *cyp11a* and *cyp17* [41]. Apparently, the results of our study further revealed the cause of MC-LR-induced endocrine disruption through endocrine axes is to interfere with steroidogenesis, including cholesterol, cortisol and estrogen. Moreover, our previous study found that exposure to MC-LR caused sex-differential gonadal impairments through disrupting the HPG-axis and growth hormone/insulin-like growth factors (GH/IGFs) systems, and the gonadal development of males was more vulnerable than that of female to MC-LR [2]. Given this information, we focused this study on male zebrafish to avoid the effects of gender differences. A further study might be needed to explore gender differences in HPI-axis activity when fish are exposed to MC-LR.

In teleost fish, the negative feedback of the HPI axis is one key mechanism that allows fish to maintain a stable status when faced with various physiological or environmental stresses [42]. Glucocorticoid receptor (GR) and mineralocorticoid receptor (MR) are the specific intracellular receptors, by which corticosteroids exert their effects [43,44]. Chen et al. [45] detected a significant down-regulation of *gr* but up-regulation of *mr* in larvae zebrafish after acute exposure to 300 μg/L MC-LR, which suggested that MC-LR acted on the expression of *gr* and *mr* and then influenced the HPI axis. Marked down-regulations of *gr* were documented in the brain of female zebrafish after exposure to 10 and 100 μg/L monocrotophos for 21 d [40]. Studies reported that mutant zebrafish with non-functional GRs displayed a loss of negative feedback when suffering from physiological stress [46,47]. Hence, in this study, the notable down-regulation of *gr* and *mr* in the brain of zebrafish treated with 25 μg/L MC-LR might suggest an inhibition on the negative feedback caused by MC-LR, which finally resulted in the elevation of cortisol. In fact, the persistent high concentration of cortisol can become a detrimental agent to the organism, referred to as a “homeostatic overload”, where cortisol itself damages organisms’ physiological stability [48]. It was reported that increased cortisol caused by toxins could affect multiple physiological processes, such as energy metabolism, growth and development, and reproduction and immunity [23,39]. Therefore, our data showed that persistent MC-LR exposure cause an extensive upregulation of HPI-axis genes but an inhibition of nuclear receptors (*gr* and *mr*), which finally increased serum cortisol levels.

Blood glucose is also used as an indirect indicator of stress in fish, since circulating glucose levels can be influenced by multiple hormones like cortisol [23,24]. For higher energy demand, the increase of cortisol could induce an elevation of the blood glucose level through the mediation of GR [23,25]. However, in the present study, the expression levels of hepatic *gr* were significantly down-regulated in all MC-LR treatment groups, which might cause the problems of downstream gene recognition and activation and lead to decreased glucose levels. Our result that serum glucose levels of fish were significantly down-regulated in all MC-LR treatments supported this hypothesis. Similarly, Woźny et al. [27] observed a significant reduction in the plasma glucose level of whitefish at 14 d after intraperitoneal injection with 100 μg/kg MC-LR of body mass. In fact, hepatic glucose hemostasis is dependent on the regulation of key metabolic enzymes and genes involved in glycogenolysis, gluconeogenesis, glycolysis and glycogenesis. Glycogenolysis and gluconeogenesis speed up the conversion of glycogen and other non-sugar substances to glucose. Among these, glycogen phosphorylase (GP) catalyzes the rate limited step in glycogen depletion, while glucose-6-phosphatase (G6Pase), fructose-1, 6-bisphosphatase (FBPase) and phosphoenolpyruvate carboxykinase (PEPCK) cause de novo glucose synthesis via gluconeogenesis. Conversely, glycolysis and glycogenesis promoted glucose oxidation and storage, which are catalyzed by glucokinase (GK), phosphofructokinase (PFK) and pyruvate kinase (PK), as well as glycogen synthase (GS) [25,49]. In the present study, along with decreased serum glucose, we detected the remarkable down-regulation of gluconeogenesis-related genes (*pepck*, *fbp1a*, *g6pca*), glycogenolysis-related gene (*pyg*), glycolysis-related genes (*gk*, *pfk1b*, *pk*) and glycogenesis-related gene (*gys2*) in the liver of fish, following long-term exposure to MC-LR, which suggested a strong suppression in hepatic glucose produce, oxygenolysis and storage. It thus appeared that the liver glucose produced was less than glucose lysis in this study, indicating that persistent MC-LR exposure caused elevated energy expenditure. Chen et al. [50] reported that exposure to 10 μg/L MC-LR for 90 d significantly decreased the expression of several genes (*pfk*, *pkl*) involved in energy metabolism in the liver of zebrafish. Zhao et al. [30] also reported a remarkable decline in the expression of gluconeogenesis rate-limiting enzyme genes, including phosphoenolpyruvate carboxykinase and glucose-6-phosphatase in mice, following the 20 μg/kg MC-LR treatment for four weeks, concomitant with the decrease of plasma glucose content. Some results on acute toxicity of MC-LR have shown that MC-LR caused damage to mitochondria and the depletion of glycogen in the liver of fish [9,28,51,52]. Mitochondria are the places that produce energy, and glycogen works as energy reserves in fish liver, so their impairments can reduce the damage repair ability of cells. Thus, the broadly restraining influences on hepatic glucose metabolism in our study revealed that long-term MC-LR exposure impaired the energy generating system, which posed a potential threat to fish. Overall, persistent exposure to MC-LR downregulated hepatic *gr*, resulting in the inhibitions of downstream genes involved in glucose metabolic enzymes and decreases in serum glucose levels. In addition, as suggested by Malbrouck et al. [51], glycogen dyshomeostasis might be the result of protein phosphatase inhibition caused by MC-LR, because glycogen synthase as a key enzyme in the glycogen synthesis pathway can be regulated by the phosphorylated form of glycogen phosphorylase in hepatocytes. Further research is required to be sure whether the decrease of circulating glucose concentration induced by MC-LR is exclusively involved with cortisol-mediated glucose metabolism abnormality or also as a result of protein phosphatase inhibition.

## 4. Conclusions

This study demonstrated that persistent MC-LR exposure caused the elevation of serum cortisol levels through modulating the expression of HPI-axis genes, which subsequently resulted in a disruption glucose hemostasis. These findings firstly established the link between persistent MC-LR exposure and impaired glucose metabolism, suggesting that long-term MC-LR-mediated stress might threaten fish’s health.

## 5. Materials and Methods

### 5.1. Chemicals and Reagents

MC-LR (purity ≥ 95%) was acquired from Express Technology Co., Ltd. (Beijing, China), the purity of which was identified by high performance liquid chromatography (HPLC). The toxin was dissolved in MilliQ water to obtain a stock solution of 0.5 mg/mL for the experiment. All of the other chemicals utilized in the experiment were of analytical grade.

### 5.2. Animal Maintenance and Experimental Design

Healthy adult male zebrafish (AB strain, 3-months-old) were purchased from the Institute of Hydrobiology, Chinese Academy of Sciences. Before the experiment, zebrafish were cultured in a closed flow-through system with 28 ± 0.5 °C and a 12 h light: 12 h dark cycle condition for a 14-d acclimation. Fish were fed with freshly hatched *Artemia* nauplii three times daily to ensure adequate nutrition. After acclimation, fish were respectively immersed into MC-LR solutions at concentrations of 0, 1, 5, and 25 μg/L for 30 d. Three replicates were performed for each concentration and each replicate was assigned randomly to 30 male adult zebrafish. Exposure concentrations were chosen, since it is relevant to the environmental concentration of MC-LR in nature water bodies [8,17,53]. In order to maintain relatively constant MC-LR concentration during exposure, 1/3 exposure solution was replaced every 3 d with fresh water, containing the corresponding MC-LR level or no MC-LR. Actual MC-LR concentrations in exposure solutions were determined every 3 d using the ELISA kit for MC-LR (Beacon Analytical System, Inc., Saco, ME, USA), with a minimum detection limit of 0.1 μg/L (data not shown).

After a 30-d exposure, zebrafish were first anesthetized with tricaine methanesulfonate (MS-222). In each exposure group, the blood samples of 10 fish were immediately collected as one replicate to separate the serum for the measurement of cortisol and glucose, and then brains, head kidneys and livers of 5 fish were sampled as one replicate for the determination of gene expression. The experiment was approved by the guidelines of Institutional Animal and Care and Use Committees (IACUC) of Huazhong Agricultural University (permission number: HZAUFI-2018-013, date of approval: 11 August 2018), Wuhan in China.

### 5.3. Serum Cortisol and Glucose Analysis

Serum cortisol was determined as described in Rance and Baker [54] using Iodine [^125^I] Cortisol Radioimmunoassay (RIA) Kit from Beijing North Institute of Biological Technology (Beijing, China). According to Trinder [55], serum glucose was tested by the GOD-POD (glucose oxidase-peroxidase) method, using a commercial kit purchased from the Shanghai Mingdian Institute of Bioengineering, Inc. (Shanghai, China).

### 5.4. Quantitative Real-Time PCR

Total RNA was respectively isolated from brain, head kidney and liver samples using RNAiso Plus reagent (TaKaRa, Dalian, China). The concentration and purity of total RNA were determined by a NanoDrop ND-2000 spectrophotometer (Thermo Scientific, Wilmington, DE, USA). According to the manufacturer’s instruction, 1 μg of total RNA was reverse transcribed for each sample by using a PrimeScript RT reagent Kit with gDNA Eraser (TaKaRa, Dalian, China). Using SYBR Green Kits (Takara, Dalian, China), quantitative real-time PCR was conducted on LightCycler^®^ 480 Real-Time PCR Detection System (Roche, Basel, Switzerland). Gene specific primers designed by Primer Premier 5.0 (Premier Biosoft International, Palo Alto, CA, USA) were listed in Table 2. The amplification protocol was set according to Lin et al. [11] In the light of our previous study [14], the housekeeping gene *gapdh* was used as an internal control for normalization. The relative expression ratio (*R*) was calculated using the 2^−ΔΔCt^ method [56]. All experiments were carried in six biological replicates.

### 5.5. Statistical Analysis

The statistical analysis was performed using SPSS 20.0 (SPSS Inc., Chicago, IL, USA) for Windows. All values were expressed as mean ± standard error (S.E.) The significant differences were evaluated by one-way analysis of variance (ANOVA), followed by Duncan’s test. Normality and variance uniformity have been verified. Statistical significance was established at *p* < 0.05.

## Figures and Tables

**Table 1:** Gene list for HPI-axis signaling and hepatic glucose metabolic pathways in male zebrafish.

Abbreviation	Gene Name
*crh*	corticotropin releasing hormone
*crhr1*	corticotropin releasing hormone receptor 1
*crhr2*	corticotropin releasing hormone receptor 2
*crhbp*	corticotropin releasing hormone binding protein
*pomc*	proopiomelanocortin
*gr*	glucocorticoid receptor
*mr*	mineralocorticoid receptor
*mc2r*	melanocortin 2 receptor
*hmgra*	hydroxy-methylglutaryl-CoA reductase a
*hmgrb*	hydroxy-methylglutaryl-CoA reductase b
*star*	steroidogenic acute regulatory protein
*cyp11a1*	cytochrome P450, family 11, subfamily A, polypeptide 1
*3β* *-hsd*	3-beta-hydroxysteroid dehydrogenase
*cyp17a1*	cytochrome P450, family 17, subfamily A, polypeptide 1
*cyp21a2*	cytochrome P450, family 21, subfamily A, polypeptide 2
*cyp11b*	cytochrome P450, family 11, subfamily B
*11β-hsd2*	11-beta-hydroxysteroid dehydrogenase-type 3
*pepck*	phosphoenolpyruvate carboxykinase
*fbp1a*	fructose-1,6-bisphosphatase 1a
*g6pca*	glucose-6-phosphatase
*gk*	glucokinase
*pfk1b*	phosphofructokinase 1b
*pk*	pyruvate kinase
*pyg*	phosphorylase, glycogen
*gys2*	glycogen synthase 2

**Table 2:** Primer sequences for genes related to HPI axis and hepatic glucose metabolism of male zebrafish.

Gene	Sequence of the Primers (5′-3′)	Accession No.	Product Length (bp)	Amplification Efficiency (%)
*crh*	F: TTCGGGAAGTAACCACAAGC	NM_001007379.1	161	109.37%
	R: CTGCACTCTATTCGCCTTCC			
*crhr1*	F: TCGGTGTTGAGTCTCATGCC	XM_691254.6	186	93.89%
	R: CGCGTGCGGGTAAAATGTAG			
*crhr2*	F: TGAACTGTTGCTCCGAGTCC	NM_001113644.1	81	101.76%
	R: GGAGAAACACGTCGCCGTTA			
*crhbp*	F: AGAGGTCCATTCTTGGCTGC	NM_001003459.1	170	98.52%
	R: GCTCGACACCATTCTGACCA			
*pomc*	F: AGGTCGACTATCCGCAAGAA	AY158003	232	97.13%
	R: CAACCTCTCCCCCTTAAAGC			
*gr*	F: TTCTACGTTGCTGACGATGC	EF567112	239	95.48%
	R: CCGGTGTTCTCCTGTTTGAT			
*mr*	F: TGCCACTACGGGGTTGTTAC	EF567113	181	102.77%
	R: GTGCCCCAAGATTCATCCCA			
*mc2r*	F: AAAGGGGTATTCGTGGCCTG	NM_180971	150	96.54%
	R: TGCCGGATCAATAACAGCGT			
*hmgra*	F: GGAACATGCACTAAGCAGGC	NM_001079977.2	143	93.24%
	R: AGAGAAGAAGGGATCGGTTGC			
*hmgrb*	F: CCGCTTCTATTGGACGGGAA	NM_001014292.2	200	90.61%
	R: AACCAGCCCTTAACCTCTGC			
*star*	F: TTTCTGGCTGGGATGTCCAC	NM_131663	105	104.15%
	R: ATCTGCACTTGGTCGCATGA			
*cyp11a*	F: TCCCGAAACCAGAGCAATAC	NM_152953	204	97.45%
	R: GCTCAAACTTGCTCCTGACC			
*3βhsd*	F: CGCACATCGTCTCAGGACAT	AY279108.1	122	106.82%
	R: GTAGAGCGCTGCGTTGAAAG			
*cyp17a1*	F: AGACCCACCACAGACCTTTAC	NM_212806.3	96	92.59%
	R: TGAGCACAATCGGCCACTTA			
*cyp21a2*	F: AATGGTCTGACTTTGCTGGGA	XM_021474355.1	222	95.38%
	R: CAGCCTTTCCACTGTAGTCTCG			
*cyp11b*	F: TGTATCCTCTGGGTCGTTCC	DQ650710	228	99.68%
	R: GCTCTTCTGTGGACGAAACC			
*11β-hsd2*	F: ATCAGAATCCATCCAGCCTTAG	NM_212720	113	92.76%
	R: ATTAGCATCCACACCAATACATC			
*pepck*	F: TGCCTGGATGAAATTTGACA	NM_213192.1	106	98.67%
	R: GGCATGAGGGTTGGTTTTTA			
*fbp1a*	F: CATCTGTATGGGATTGCTGG	NM_199942	173	93.12%
	R: TTACCCCGTCTATCTGGCTC			
*g6pca*	F: GGCTGAACCTCGTCCTAAAGT	BC076446	203	93.11%
	R: GATTGAAAGCAACGCTGTGAT			
*gk*	F: TGAGGATGAAGAGCGAGGC	BC122359	178	91.9%
	R: AGAGAAGGTGAATCCCAGCG			
*pfk1b*	F: GCATGTGTGTCATTCCTGCC	NM_001328389.1	194	104.5%
	R: GCCTGTAGTGGTTGCCAGAT			
*pk*	F: AGAAACAGCCAAAGGACA	BC152219	253	103.16%
	R: ACGAGGACGATAACGAGA			
*pyg*	F: GAAAGGTTTCAACCGGCACC	NM_001008538.1	88	90.8%
	R: GTGCGACAGCGCGAAATAAT			
*gys2*	F: GTGCGATGCGACTATCCAGA	NM_001018679.1	188	106.6%
	R: TTCACCCCATTCGTCCACAG			
*gapdh*	F: CTGGTGACCCGTGCTGCTT	NM_001115114	150	98.2%
	R: TTTGCCGCCTTCTGCCTTA			

## References

[1] Li L., Xie P., Lei H., Zhang X. (2013). Renal accumulation and effects of intraperitoneal injection of extracted microcystins in omnivorous crucian carp (*Carassius auratus*). Toxicon.

[2] Hou J., Su Y., Lin W., Guo H., Xie P., Chen J., Gu Z., Li L. (2017). Microcystin-LR retards gonadal maturation through disrupting the growth hormone/insulin-like growth factors system in zebrafish. Ecotoxicol. Environ. Saf..

[3] Rezaitabar S., Sari A.E., Bahramifar N., Ramezanpour Z. (2017). Transfer, tissue distribution and bioaccumulation of microcystin-LR in the phytoplanktivorous and carnivorous fish in Anzali wetland, with potential health risks to humans. Sci. Totol. Environ..

[4] Bouaïcha N., Miles C.O., Beach D.G., Labidi Z., Djabri A., Benayache N.Y., Nguyen-Quang T. (2019). Structural diversity, characterization and toxicology of microcystins. Toxins.

[5] Buratti F.M., Manganelli M., Vichi S., Stefanelli M., Scardala S., Testai E., Funari E. (2017). Cyanotoxins: Producing organisms, occurrence, toxicity, mechanism of action and human health toxicological risk evaluation. Arch. Toxicol..

[6] World Health Organization (1998). Health Organization. Health criteria and other supporting information: Addendum. Guidelines for Drinking-Water Quality.

[7] Chen J., Xie P., Li L., Xu J. (2009). First identification of the hepatotoxic microcystins in the serum of a chronically exposed human population together with indication of hepatocellular damage. Toxicol. Sci..

[8] Wang Q., Niu Y., Xie P., Chen J., Ma Z., Tao M., Qi M., Wu L., Guo L. (2010). Factors affecting temporal and spatial variations of microcystins in Gonghu Bay of Lake Taihu, with potential risk of microcystin contamination to human health. Sci. World J..

[9] Li L., Xie P., Chen J. (2005). In vivo studies on toxin accumulation in liver and ultrastructural changes of hepatocytes of the phytoplanktivorous bighead carp ip-injected with extracted microcystins. Toxicon.

[10] Li D., Xie P., Zhang X., Zhao Y. (2009). Intraperitoneal injection of extracted microcystins results in hypovolemia and hypotension in crucian carp (*Carassius auratus*). Toxicon.

[11] Lin W., Hou J., Guo H., Qiu Y., Li L., Li D., Tang R. (2017). Dualistic immunomodulation of sub-chronic microcystin-LR exposure on the innate-immune defense system in male zebrafish. Chemosphere.

[12] Li G., Cai F., Yan W., Li C., Wang J. (2012). A proteomic analysis of MCLR-induced neurotoxicity: Implications for Alzheimer’s disease. Toxicol. Sci..

[13] Zhao Y., Xie L., Yan Y. (2015). Microcystin-LR impairs zebrafish reproduction by affecting oogenesis and endocrine system. Chemosphere.

[14] Hou J., Li L., Wu N., Su Y., Lin W., Li G., Gu Z. (2016). Reproduction impairment and endocrine disruption in female zebrafish after long-term exposure to MC-LR: A life cycle assessment. Environ. Pollut..

[15] Liu W., Chen C., Chen L., Wang L., Li J., Chen Y., Jin J., Kawan A., Zhang X. (2016). Sex-dependent effects of microcystin-LR on hypothalamic-pituitary-gonad axis and gametogenesis of adult zebrafish. Sci. Rep..

[16] Su Y., Li L., Hou J., Wu N., Lin W., Li G. (2016). Life-cycle exposure to microcystin-LR interferes with the reproductive endocrine system of male zebrafish. Aquat. Toxicol..

[17] Hou J., Su Y., Lin W., Guo H., Li L., Anderson D.M., Li D., Tang R., Chi W., Zhang X. (2018). Estrogenic potency of MC-LR is induced via stimulating steroidogenesis: In vitro and in vivo evidence. Environ. Pollut..

[18] Liu Z., Li D., Hu Q., Tang R., Li L. (2016). Effects of exposure to microcystin-LR at environmentally relevant concentrations on the metabolism of thyroid hormones in adult zebrafish (*Danio rerio*). Toxicon.

[19] Cheng H., Yan W., Wu Q., Liu C., Gong X., Hung T.C., Li G. (2017). Parental exposure to microcystin-LR induced thyroid endocrine disruption in zebrafish offspring, a transgenerational toxicity. Environ. Pollut..

[20] Liu C., Zhang X., Deng J., Hecker M., Al-Khedhairy A., Giesy J.P., Zhou B. (2011). Effects of prochloraz or propylthiouracil on the cross-talk between the HPG, HPA, and HPT axes in zebrafish. Environ. Sci. Technol..

[21] Bury N.R., Eddy F.B., Codd G.A. (1996). Stress responses of brown trout, *Salmo trutta* L., to the cyanobacterium, *Microcystis aeruginosa*. Environ. Toxicol. Water Qual..

[22] Li D., Xie P., Zhang X. (2008). Changes in plasma thyroid hormones and cortisol levels in crucian carp (*Carassius auratus*) exposed to the extracted microcystins. Chemosphere.

[23] Mommsen T.P., Vijayan M.M., Moon T.W. (1999). Cortisol in teleosts: Dynamics, mechanisms of action, and metabolic regulation. Rev. Fish Biol. Fish..

[24] Trenzado C.E., Carrick T.R., Pottinger T.G. (2003). Divergence of endocrine and metabolic responses to stress in two rainbow trout lines selected for differing cortisol responsiveness to stress. Gen. Comp. Endocrinol..

[25] López-Patiño M.A., Hernández-Pérez J., Gesto M., Librán-Pérez M., Míguez J.M., Soengas J.L. (2014). Short-term time course of liver metabolic response to acute handling stress in rainbow trout, *Oncorhynchus mykiss*. Comp. Biochem. Phys. A.

[26] Ernst B., Hoeger S.J., O’Brien E., Dietrich D.R. (2006). Oral toxicity of the microcystin-containing cyanobacterium *Planktothrix rubescens* in European whitefish (*Coregonus lavaretus*). Aqua. Toxicol..

[27] Woźny M., Lewczuk B., Ziółkowska N., Gomułka P., Dobosz S., Łakomiak A., Florczyk M., Brzuzan P. (2016). Intraperitoneal exposure of whitefish to microcystin-LR induces rapid liver injury followed by regeneration and resilience to subsequent exposures. Toxicol. Appl. Pharm..

[28] Paulino M.G., Rossi P.A., Venturini F.P., Tavares D., da Silva Souza N.E., Sakuragui M.M., Moraes G., Terezan A.P., Fernandes J.B., Giani A. (2017). Hepatotoxicity and metabolic effects of cellular extract of cyanobacterium *Radiocystis fernandoi* containing microcystins RR and YR on neotropical fish (*Hoplias malabaricus*). Chemosphere.

[29] Mezhoud K., Praseuth D., Puiseux-Dao S., Francois J.C. (2008). Global quantitative analysis of protein expression and phosphorylation status in the liver of the medaka fish (*Oryzias latipes*) exposed to microcystin-LR, Balneation study. Aquat. Toxicol..

[30] Zhao S., Li G., Chen J. (2015). A proteomic analysis of prenatal transfer of microcystin-LR induced neurotoxicity in rat offspring. J. Proteom..

[31] Moon T.W. (2001). Glucose intolerance in teleost fish: Fact or fiction?. Comp. Biochem. Phys. B.

[32] Alsop D., Vijayan M.M. (2009). Molecular programming of the corticosteroid stress axis during zebrafish development. Comp. Biochem. Phys. A.

[33] Alsop D., Vijayan M. (2009). The zebrafish stress axis: Molecular fallout from the teleost-specific genome duplication event. Gen. Comp. Endocrinol..

[34] Liu C., Yu H., Zhang X. (2013). Zebrafish embryos/larvae for rapid determination of effects on hypothalamic-pituitary-thyroid (HPT) and hypothalamic-pituitary-interrenal (HPI) axis: mRNA expression. Chemosphere.

[35] Alderman S.L., Bernier N.J. (2009). Ontogeny of the corticotropin-releasing factor system in zebrafish. Gen. Comp. Endocrinol..

[36] Wu Q., Yan W., Liu C.S., Li L., Yu L.Q., Zhao S.J., Li G.Y. (2016). Microcystin-LR exposure induces developmental neurotoxicity in zebrafish embryo. Environ. Pollut..

[37] Yan W., Zhou Y., Yang J., Li S., Hu D., Wang J., Chen J., Li G. (2012). Waterborne exposure to microcystin-LR alters thyroid hormone levels and gene transcription in the hypothalamic–pituitary–thyroid axis in zebrafish larvae. Chemosphere.

[38] Liu Z., Tang R., Li D., Hu Q., Wang Y. (2015). Subacute microcystin-LR exposure alters the metabolism of thyroid hormones in juvenile zebrafish (*Danio Rerio*). Toxins.

[39] Fuzzen M.L.M., Van Der Kraak G., Bernier N.J. (2010). Stirring up new ideas about the regulation of the hypothalamic-pituitary-interrenal axis in zebrafish (*Danio rerio*). Zebrafish.

[40] Zhang X., Zhong Y., Tian H., Wang W., Ru S. (2015). Impairment of the cortisol stress response mediated by the hypothalamus–pituitary–interrenal (HPI) axis in zebrafish (*Danio rerio*) exposed to monocrotophos pesticide. Comp. Biochem. Phys. C.

[41] Arukwe A. (2005). Modulation of barin steroidogenesis by affecting transcriptional changes of steroidogenic acute regulatory (StAR) protein and cholesterol side chain cleavage (P450scc) in juvenile Atlantic salmon (*Salmo salar*) is a novel aspect of nonylphenol toxicity. Environ. Sci. Technol..

[42] Nichols J.W., Breen M., Denver R.J., DiStefano J.J., Edwards J.S., Hoke R.A., Volz D.C., Zhang X. (2011). Predicting chemical impacts on vertebrate endocrine systems. Environ. Toxicol. Chem..

[43] Bury N.R., Sturm A. (2007). Evolution of the corticosteroid receptor signaling pathway in fish. Gen. Comp. Endocrinol..

[44] Schaaf M.J.M., Chatzopoulou A., Spaink H.P. (2009). The zebrafish as a model system for glucocorticoid receptor research. Comp. Biochem. Phys. A.

[45] Chen L., Wang Y., Giesy J.P., Chen F., Shi T., Chen J., Xie P. (2018). Microcystin-LR affects the hypothalamic-pituitary-inter-renal (HPI) axis in early life stages (embryos and larvae) of zebrafish. Environ. Pollut..

[46] Griffiths B., Schoonheim P.J., Ziv L., Voelker L., Baier H., Gahtan E. (2012). A zebrafish model of glucocorticoid resistance shows serotonergic modulation of the stress response. Front. Behav. Neurosci..

[47] Ziv L., Muto A., Schoonheim P.J., Meijsing S.H., Strasser D., Ingraham H.A., Schaaf M.J.M., Yamamoto K.R., Baier H. (2013). An affective disorder in zebrafish with mutation of the glucocorticoid receptor. Mol. Psychiatry.

[48] Romero L.M., Dickens M.J., Cyr N.E. (2009). The reactive scope model—A new model integrating homeostasis, allostasis, and stress. Horm. Behav..

[49] Zhao F., Jiang G., Wei P., Wang H., Ru S. (2018). Bisphenol S exposure impairs glucose homeostasis in male zebrafish (*Danio rerio*). Ecotoxicol. Environ. Saf..

[50] Chen L., Hu Y., He J., Chen J., Giesy J.P., Xie P. (2016). Responses of the proteome and metabolome in livers of zebrafish exposed chronically to environmentally relevant concentrations of microcystin-LR. Environ. Sci. Technol..

[51] Malbrouck C., Trausch G., Devos P., Kestemont P. (2004). Effect of microcystin-LR on protein phosphatase activity and glycogen content in isolated hepatocytes of fed and fasted juvenile goldfish *Carassius auratus* L. Toxicon.

[52] Hou J., Li L., Xue T., Long M., Su Y., Wu N. (2015). Hepatic positive and negative antioxidant responses in zebrafish after intraperitoneal administration of toxic microcystin-LR. Chemosphere.

[53] Lin W., Hou J., Guo H., Li L., Wang L., Zhang D., Li D., Tang R. (2018). The synergistic effects of waterborne microcystin-LR and nitrite on hepatic pathological damage, lipid peroxidation and antioxidant responses of male zebrafish. Environ. Pollut..

[54] Rance T.A., Baker B.I. (1981). The in vitro response of the trout interrenal to various fragments of ACTH. Gen. Comp. Endocrinol..

[55] Trinder P. (1969). Determination of glucose in blood using glucose oxidase with an alternative oxygen acceptor. Ann. Clin. Biochem..

[56] Livak K.J., Schmittgen T.D. (2001). Analysis of relative gene expression data using real-time quantitative PCR and the 2^−ΔΔCT^ method. Methods.

